# Metabolomic Study of Collagen-Induced Arthritis in Rats and the Interventional Effects of Huang-Lian-Jie-Du-Tang, a Traditional Chinese Medicine

**DOI:** 10.1155/2013/439690

**Published:** 2013-03-06

**Authors:** Rongcai Yue, Ling Zhao, Yaohua Hu, Peng Jiang, Shuping Wang, Li Xiang, Wencong Liu, Lei Shan, Weidong Zhang, Runhui Liu

**Affiliations:** ^1^School of Pharmacy, Second Military Medical University, 325 Guo-He Road, Shanghai 200433, China; ^2^College of Traditional Chinese Medicine, Jilin Agricultural University, Changchun 130118, China; ^3^School of Pharmacy, Shanghai Jiao Tong University, Shanghai 200030, China

## Abstract

Huang-Lian-Jie-Du-Tang (HLJDT) is a traditional Chinese medicine (TCM) with anti-inflammatory activity. The present study used a metabolomic approach based on LC-Q-TOF-MS to profile rheumatoid-arthritis- (RA-) related metabolic changes and to investigate the interventional mechanisms of HLJDT in collagen-induced arthritis rats. Forty male Wistar rats were randomly divided into five groups: (1) a model group, (2) a normal control group, (3) a dexamethasone group, (4) a HLJDT group, and (5) a group that received 13 components of HLJDT. Plasma samples were collected 8, 15, and 22 days after the rats were injected with bovine type II collagen. By combining variable importance in the projection values with partial least squares discriminant analysis, 18 potential biomarkers were identified in the plasma samples. The biomarkers were primarily involved in glycerophospholipid metabolism, fatty acid metabolism, tryptophan metabolism, linoleic acid metabolism, phenylalanine metabolism, purine metabolism, arachidonic acid metabolism, and bile acid biosynthesis. Using the potential biomarkers as a screening index, the results suggest that HLJDT can potentially reverse the process of RA by partially regulating fatty acid oxidation and arachidonic acid metabolism. This study demonstrates that a metabolomic strategy is useful for identifying potential RA biomarkers and investigating the underlying mechanisms of a TCM in RA treatment.

## 1. Introduction

Rheumatoid arthritis (RA) is an autoimmune disease characterized by persistent synovitis, systemic inflammation, and autoantibodies [1, 2]. RA primarily affects the small diarthrodial joints of the hands and feet, causing swelling and pain. If left untreated, RA may result in deformity. Several studies have investigated the pathogenetic mechanisms of RA, particularly collagen-induced arthritis (CIA) in humans and in animal models [3–5]. Although some important biomarkers and novel therapeutic methods have been identified and developed for RA diagnosis and treatment [6–8], the occurrence of RA-induced deformity remains high. Moreover, current RA treatment medications are limited by several well-characterized clinical side effects, such as hepatotoxicity [9, 10], gastrointestinal effects [11], and cardiotoxic effects [12]. Therefore, further investigation of the biological processes related to RA, as well as its clinical diagnosis and treatment, is needed to understand its disease mechanism, identify new biomarkers, and explore new anti-RA drugs.

Metabolomics focuses on the comprehensive measurement of all small molecular weight compounds, including endogenous and exogenous species, which are present in a biological system. Furthermore, it provides a functional readout of abnormal, disease-related physiological states in the human body and may provide new insights into the global effects of disease related to metabolic pathways [13]. In recent years, metabolomics has been used to identify disease-related biomarkers and shows significant potential for the early diagnosis, therapeutic monitoring and pathogenic understanding of many diseases, including RA [14–16]. Among the analytical techniques used in metabolomic research, LC-MS is recognized as one of the most selective, sensitive, and reproducible methods [17] due to its enhanced reproducibility of retention times [18]. This reproducibility is especially important for large-scale, untargeted metabolic profiling.

Huang-Lian-Jie-Du-Tang is an aqueous extract that consists of four herbal materials: *Rhizoma Coptidis*, *Radix Scutellariae*, *Cortex Phellodendri*, and *Fructus Gardeniae*. In our previous study, LC-DAD and LC-ESI-MS methods were developed and validated for the chromatographic fingerprinting and quantitative analysis of HLJDT [19]. Furthermore, HLJDT's potentially active components were identified in a plasma-based pharmacochemical study [20]. In addition, the anti-inflammatory activities, component herbs and active components of HLJDT were also investigated [21, 22]. However, a proper approach for evaluating the holistic efficacy of such a multicomponent medicine is urgently needed. In the present study, we used a metabolomic approach to investigate the biochemical abnormalities associated with RA and to assess the therapeutic effects of HLJDT and its components in CIA rats. 

## 2. Experimental Methods

### 2.1. Reagents and Materials

HPLC-grade methanol and acetonitrile were purchased from J.T. Baker (NJ, USA). Ultrapure water (18.2 MΩ) was prepared with a Milli-Q water purification system (Millipore, MA, USA). The following HLJDT components were purchased from the National Institute for the Control of Pharmaceutical and Biological Products (Beijing, China): geniposide, coptisine, phellodendrine, jatrorrhizine, magnoflorine, palmatine, berberine, baicalin, chlorogenic acid, crocin, wogonoside, baicalein, and wogonin. The following standard metabolites were obtained from Sigma-Aldrich (St. Louis, MO, USA): choline, carnitine, L-phenylalanine, arachidonic acid, hippuric acid, uric acid, allantoin, 5-hydroxy tryptophan, and L-tryptophan.

*Rhizoma Coptidis* (*Rhizoma* of *Coptis chinensis* Franch.), *Radix Scutellariae* (*Radix* of *Scutellaria baicalensis* Georgi.), *Cortex Phellodendri* (*Cortex* of *Phellodendron chinense* Schneid.), and *Fructus Gardeniae* (*Fructus* of *Gardenia jasminoides* Ellis.) were purchased from Bozhou (Anhui province, China) and were authenticated by Professor HanMing Zhang (Second Military Medical University, Shanghai, China). Voucher specimens of *Rhizoma coptidis*, *Radix scutellariae*, *Cortex phellodendri*, and *Fructus gardeniae* were stored at the Second Military Medical University, Shanghai, China (no. HL20070523, HQ20070523, HB20070523, and ZZ20070523, resp.). HLJDT extract was prepared from the four medicinal herbs (*Rhizoma Coptidis*, *Radix Scutellariae*, *Cortex Phellodendri*, and *Fructus Gardeniae* in a 3 : 2 : 2 : 3 ratio) as previously described in [19, 20]. All TCM mixtures were maintained under careful quality control to ensure their identification throughout the experiments.

### 2.2. Animals

Adult male Wistar rats (140–160 g) were purchased from the SLAC Laboratory Animal Co. (Shanghai, China). Rats were kept in SPF-grade Experimental Animal Houses (the Second Military Medical University, Shanghai) with free access to food and water under standard temperature conditions (22°C) and a 12 h light/dark cycle. The animal experiments were conducted in strict accordance to the National Institutes of Health's Guide to the Care and Use of Laboratory Animals. The animal experiments were approved by the local institutional review board at the authors' affiliated institutions.

### 2.3. CIA Model and Drug Administration

Type II collagen (Chondrex, Redmond, WA, USA) was emulsified with incomplete Freund's adjuvant at a 1 : 1 ratio. Rats were intradermally injected with 2 mg/kg of collagen-IFA suspension at the base of the tail (day 0). A boost injection with 1 mg/kg of the collagen-IFA suspension was given on day 7 in the same manner.

Forty rats were randomly divided into 5 groups of 8 rats each: (1) rats without CIA immunization (normal control group, NG), (2) rats with CIA immunization (CIA model group, MG), (3) CIA rats treated with 270 mg/kg of HLJDT (HLJDT group, HG), (4) CIA rats treated with the 13 main components of HLJDT (components group, CG), and (5) rats treated with 0.05 mg/kg of dexamethasone (Sine Phama Lab Co., Ltd., Shanghai, China) (positive control group, DG). A dry powder of HLJDT was dissolved in 0.5% carboxymethyl cellulose sodium (CMC-Na), stirred at 37°C for 1 h and administered orally to the CIA rats. This 270 mg/kg dose was explored in the animal experiment and is considered within the MTD (2 g/kg) for oral administration. Based on the quantitative analysis of HLJDT [19] and a dosage of 270 mg/kg of HLJDT, 13 main components were identified: 5.67 mg/kg of geniposide, 0.24 mg/kg of coptisine, 0.35 mg/kg of phellodendrine, 1.2 mg/kg of jatrorrhizine, 1.31 mg/kg of magnoflorine, 2.07 mg/kg of palmatine, 12.97 mg/kg of berberine, 10.55 mg/kg of baicalin, 1.05 mg/kg of chlorogenic acid, 0.39 mg/kg of crocin, 2.39 mg/kg of wogonoside, 1.45 mg/kg of baicalein, and 0.88 mg/kg of wogonin. The components were mixed, dissolved in 0.5% CMC-Na solution, and administered intragastrically to the CIA rats. The NG and MG rats received oral administrations of an equal volume of 0.5% CMC-Na aqueous solution. All of the drug treatments were administered daily from day 0 to 28.

### 2.4. Assessment of Arthritis in Rats

After the second immunization, the rats were checked for the development of arthritis based on the extent of edema and/or erythema in their paws. The incidence and severity of arthritis were evaluated by observing changes in their arthritis scores every 2 days, measuring hind paw volumes every 4 days and measuring body weight every 3 days (only when arthritic signs were present). The observed severity of the arthritis was assessed by a semiqualitative score as follows: 0, normal, with no macroscopic signs of arthritis or swelling; 1, mild but distinct redness and swelling of the ankle or apparent redness and swelling of the individual digits, regardless of the number of affected digits; 2, moderate redness and swelling of the ankle; 3, redness and swelling of the entire paw, including the digits; and 4, maximally inflamed limb with the involvement of multiple joints. In these studies, the maximum score was 8, which represents the sum of the scores of both hind paws in each animal. The hind paw volumes were measured with a plethysmometer (7140UGO, Basile, Comerio, Italy) and were recorded as the mean volume displacement of both hind paws in each rat. A precision balance (Sartorius AG, Goettingen, Germany) was used to monitor changes in body weight.

### 2.5. Lipid Peroxide Assay and Antioxidant Enzyme Activity Assays

The plasma samples were obtained by centrifuging blood samples for 10 min at 3500 rpm and 4°C. The supernatant was used in the subsequent bioassays. Malondialdehyde (MDA), superoxide dismutase (SOD), and glutathione peroxidase (GSH-Px) assays were performed using commercially available kits according to their manufacturer's instructions (Jiancheng Bioengineering Institute, Nanjing, China). Briefly, the lipid peroxide content was determined by measuring the concentration of thiobarbituric-acid- (TBA-) reactive substances. The TBA-reactive content was expressed in terms of MDA content using l,l,3,3-tetraethoxypropane as a standard. The absorbance was measured at 532 nm and the values were expressed as nmol of MDA per mg of protein. The SOD assay was based on SOD's inhibitory effects on the spontaneous autoxidation of 6-hydroxydopamine. One IU of SOD is required to inhibit the initial rate of 6-hydroxydopamine autoxidation by 50%. The GSH-Px activity assay is based on measurements of decreasing absorbance at 340 nm due to the consumption of NADPH.

### 2.6. Sample Preparation

The plasma samples were collected from the NG, MG, DG, HG, and CG on days 8, 15, and 22. The samples were stored at −80°C prior to analysis. One-hundred-microliter aliquots of plasma were diluted with 300 *μ*L of methanol. After vortex-mixing the solution for 1 min and centrifuging it at 12000 rpm for 10 min, the supernatant was transferred to autosampler vials. A quality control (QC) sample was prepared by mixing 20 *μ*L aliquots from each group with plasma and handled in the same manner described above. The QC sample was used for monitoring the stability of sequence analysis and was continuously analyzed 6 times to validate the repeatability of the equipment.

### 2.7. LC-Q-TOF-MS Conditions

LC-Q-TOF-MS analysis was performed on an Agilent-1290 LC system (Agilent Technologies, Palo Alto, CA, USA) coupled with an electrospray ionization (ESI) source and an Agilent-6530 Q-TOF mass spectrometer. Chromatographic separation was performed on a Zorbax SB-C18 column (1.8 *μ*m, 2.1 mm × 150 mm, Agilent) with the column temperature set at 40°C. Ultrapure water with 0.1% formic acid (A) and acetonitrile (B) was used in the mobile phase according to the following gradient program: 0–2 min, 5% B; and 2–5 min, 5–50% B; and 5-6 min, 50% B; 6–17 min, 50–95% B; followed by a 5-min reequilibration step. The mobile phase flow rate was 0.3 mL/min, and the sample injection volume was 4 *μ*L.

Positive and negative ion modes were used in mass detection. The source parameters were set as follows: drying gas flow rate, 11 L/min; gas temperature, 350°C; pressure of nebulizer gas, 45 psig; Vcap, 4000 V in positive mode and 3000 V in negative mode; fragmentor, 120 V; skimmer, 45 V; and scan range, *m/z* 50–1000. The MS/MS analysis was acquired in targeted MS/MS mode with the collision energy ranging from 10 V to 40 V. 

### 2.8. Data Processing, Multivariate Data Analysis and Biomarker Identification

The MS spectra were processed using Agilent's Mass Hunter Qualitative Analysis Software (Version B.03.01, Agilent Technologies, USA) for peak detection. A list of detected peak intensities was generated using the retention time *m/z* data pairs as identifiers. The resultant normalized peak intensities formed a single matrix with retention time *m/z* pairs for each file in the data set. All of the processed data were normalized and scaled for each chromatogram prior to multivariate statistical analysis. Integrated raw mass spectrometric data were processed using Agilent's Mass Profiler Software (Version B.02.00, Agilent Technologies, USA). The intensity of each ion was normalized with respect to the total ion count to generate a data matrix consisting of the retention time, the *m/z* value, and the normalized peak area. The ion intensity of each peak was normalized to 10,000 and to the sum of its peak intensities within the sample. The processed data were exported and further processed by PCA and PLS-DA using the SIMCA-P software package (Version 11, Umetrics AB, Umeå, Sweden). The data were processed by unit variance scaling and were mean-centered using the SIMCA-P software. Model quality was evaluated based on the relevant values of *R* ^2^ and *Q* ^2^. Potential markers of interest were extracted from the values of variable importance in the projections (VIP > 1), which were constructed from PLS-DA analysis. *P* values were obtained from Student's *t*-test (*P* < 0.05). The exact molecular mass data from redundant *m/z* peaks, which correspond to the formation of different parent and product ions, were used to confirm the molecular mass of the metabolites. MS/MS data analysis highlighted neutral losses or product ions, which are characteristic of metabolite groups and can be used to discriminate between database hits. The identities of specific metabolites were confirmed by comparing their mass spectra and chromatographic retention times to commercially available reference standards. The metabolites were also identified at the Scripps Center for Metabolomics and Mass Spectrometry (METLIN). The biochemical reactions associated with these metabolites were obtained from the Kyoto Encyclopedia of Genes and Genomes (KEGG) and the Human Metabolome Database (HMDB). The fold changes were calculated as Fold = log_2_ (average peak intensity of group A/average peak intensity of group B).

### 2.9. Statistical Analysis

All quantitative data were expressed as the mean ± SD as indicated. The comparisonsbetween the two groups were analyzed by an unpaired Student *t*-test and multiple comparisons were analyzed by one-way analysis of variance (ANOVA) followed by Tukey's HSD post hoc test. Statistical significance was established as *P* < 0.05.

## 3. Results and Discussion

### 3.1. Assessment of the CIA Model

**Figure 1:**
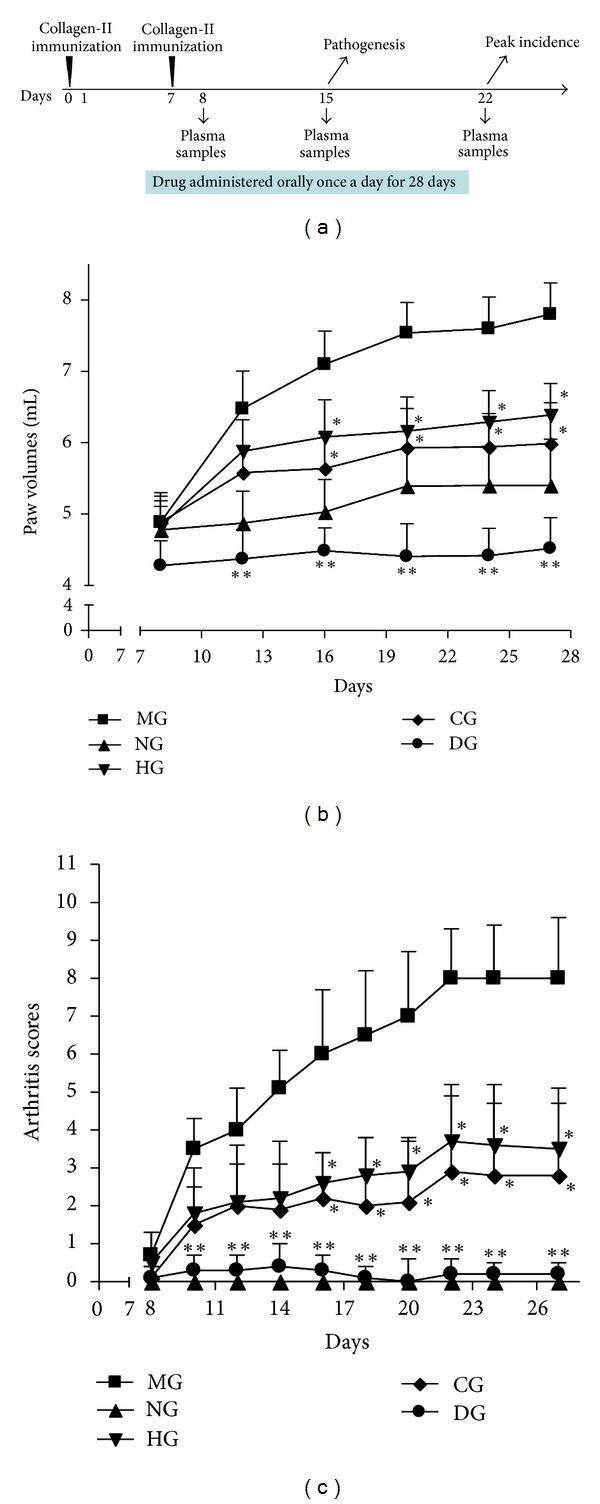
(a) Time schedule for CIA immunization, pathogenesis, peak incidence, drug administration, and sample collection for examinations. Wistar rats were immunized with bovine type II collagen with incomplete Freund's adjuvant and randomly divided into normal control, model, dexamethasone (0.05 mg/kg), HLJDT (270 mg/kg), and its 13-component groups on the day of arthritis onset (day 0, *n* = 8). (b) Hind paw volumes of each rat were evaluated every 4 days. Oral treatment of CIA rats with dexamethasone (0.05 mg/kg), HLJDT (270 mg/kg), and its components daily significantly ameliorated the severity and development of arthritis from day 16 (*P* < 0.05 versus model control). (c) Arthritis was scored every 2 days. Oral treatment of CIA rats with dexamethasone (0.05 mg/kg), HLJDT (270 mg/kg), and its components daily significantly reduced arthritis from day 16 (*P* < 0.01, *P* < 0.05, and *P* < 0.05 versus model group, resp.). NG, normal control group; MG, model group; DG, dexamethasone group; HG, HLJDT group; CG, components group.

Immunization with bovine type II collagen (coadministered with incomplete Freund's adjuvant) started producing severe arthritis 10 days after primary immunization and reached a peak on day 22 in the model group (Figure 1(a)). The decrease of arthritis in CIA rats that were treated with HLJDT and its components was further examined. Compared to the model group, swollen paws were significantly reduced in the dexamethasone, HLJDT, and component groups: (*P* < 0.05) (Figure 1(b)). Furthermore, after type II collagen immunization, the arthritis scores of CIA rats in the dexamethasone, HLJDT and components groups were significantly lower than those in the model group on days 16–22 (*P* < 0.01, *P* < 0.05, and *P* < 0.05 versus model group, resp.) (Figure 1(c)). 

### 3.2. Effects of HLJDT and Its Components on Lipid Peroxide and Antioxidant Enzyme Activities

Immunization with bovine type II collagen caused a significant decrease in the activities of SOD (2.95 ± 0.22 versus 4.56 ± 0.24, *P* < 0.01) and GSH-Px (4.97 ± 1.26 versus 7.72 ± 0.71, *P* < 0.05) and a significant increase in MDA levels (4.98 ± 0.53 versus 2.71 ± 0.34, *P* < 0.01) in comparison to the normal group on day 22 (Table 1). Compared to the test groups, the administration of HLJDT and its components caused an increase in SOD and GSH-Px activity and a decrease in MDA levels in the model control group. These findings indicate that HLJDT and its components possess potent antioxidant activities in CIA rats.

### 3.3. Assessment of the Repeatability and Stability of the LC-Q-TOF-MS Method

The repeatability and stability of the LC-Q-TOF-MS method were validated by analyzing 6 injections of identical QC samples that were prepared according to the same protocol. The relative standard deviations of the peak retention times and areas were less than 1.0% and 5.0%, respectively. Thus, the precision and repeatability of the proposed method were satisfactory for metabolomic analysis.

**Figure 2:**
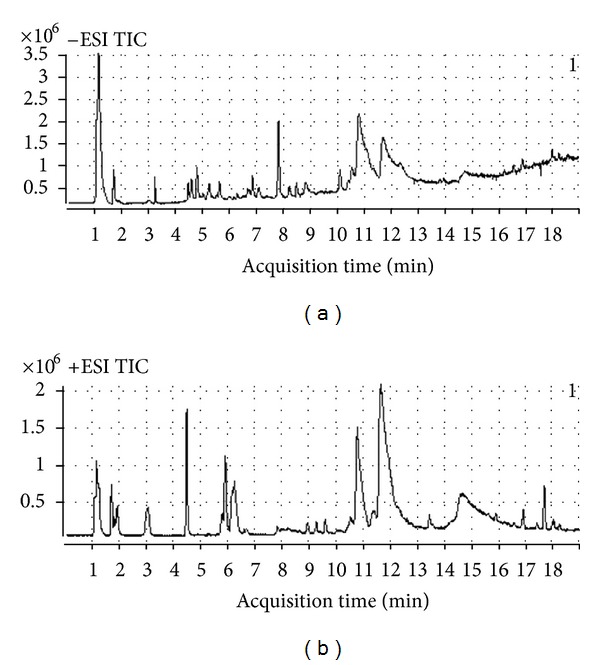
Representative base peak intensity chromatogram of the rat plasma obtained in ESI negative mode (a) and ESI positive mode (b) based on LC-Q-TOF-MS.

Fingerprints of the plasma samples were acquired in positive and negative modes. After comparing our results between both nodes, we observed higher noise, fewer peaks, and a matrix effect in the negative mode, whereas the total ionic chromatogram (TIC) of the positive mode was more suitable for analysis (Figure 2). Moreover, most of the metabolites that were detected in the plasma samples were less polar than those observed in the urine samples described in our previous study [23].

### 3.4. Multivariate Statistical Analysis and Potential Biomarker Identification

**Figure 3:**
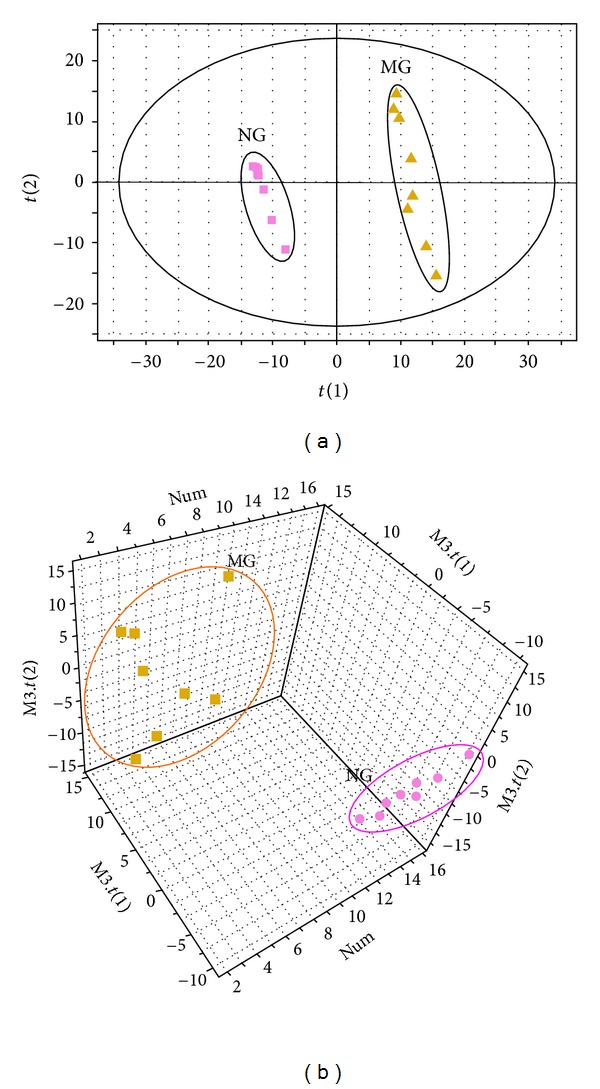
Results of multiple pattern recognition of plasma biomarkers between normal control group and model group on day 22. PLS-DA score plot (*R* ^2^ *X* = 0.253, *R* ^2^ *Y* = 0.997, *Q*_2_ = 0.875, *n* = 8) of NG and MG. NG, normal control group; MG, model group.

Ions were generated in the LC-Q-TOF-MS analysis. PLS-DA, a supervised method, is frequently used to classify groups that show metabolic differences and to extract potential biomarkers. After PLS-DA processing, the CIA model group was clearly separated from the normal control group on day 22 (Figure 3). Variables were also generated based on the values of variable importance in the projection (VIP > 1). Then, by combining Student's *t*-test with the selected variables, distinct metabolites were identified (*P* < 0.05) and selected for further study. 

**Figure 4:**
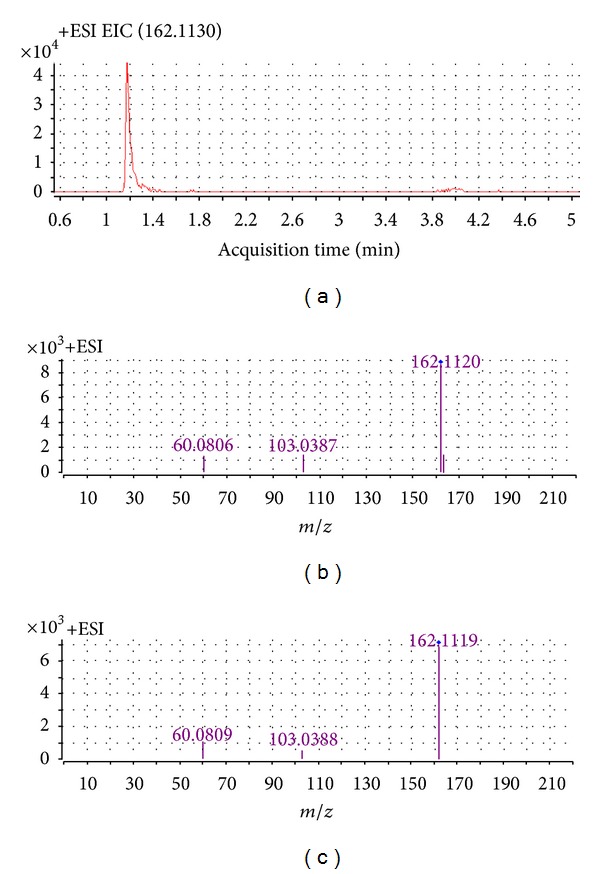
Identification of a selected biomarker (*m/z* 162.1129). (a) Extracted ion chromatogram (EIC) of *m/z* 162.113 (*t*_*R*_ = 1.19 min); (b) MS/MS spectrum of the ion; (c) MS/MS spectrum of a commercial standard carnitine. The collision energy was 10 V.

The three steps to identify these biomarkers were as follows. First, the MS^2^ spectrum of significantly different metabolic ions was obtained using a targeted MS/MS mode. Next, several online databases, such as METLIN (http://metlin.scripps.edu/), HMDB (http://www.hmdb.ca/), and KEGG (http://www.kegg.jp/), were used for initial determination of the markers. Finally, the metabolites were compared to the standard MS^2^ spectrum (see Figure 4 for an example using carnitine at *m/z* 162 to illustrate the identification process). 

**Figure 5:**
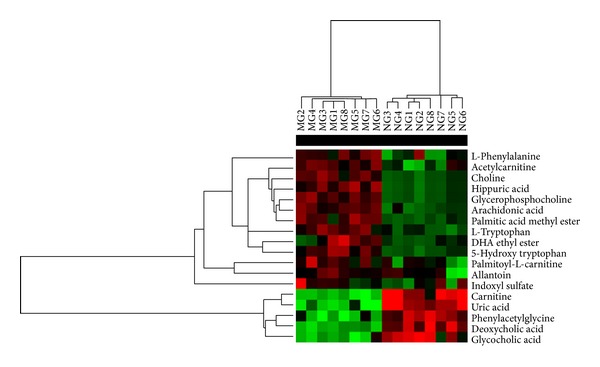
All the potential biomarkers in response to RA detected by cluster analysis. The columns show the expression levels and each row represents a biomarker. The red color indicates upregulated biomarkers compared with normal control group, while the green color represents downregulated biomarkers compared with normal control group. NG, normal control group; MG, model group; *n* = 8.

Following the identification process, 18 unique metabolites were identified (Table 2), including 11 identified in the positive mode and 7 identified in the negative mode. Three of the metabolites (L-phenylalanine, allantoin, and indoxyl sulfate) were repeatedly detected in the urine samples described in our previous study. Furthermore, 13 metabolites were upregulated, and 5 metabolites were downregulated in the model group compared to the normal control group (Figure 5). These metabolites were mainly associated with glycerophospholipid metabolism, fatty acid metabolism, tryptophan metabolism, linoleic acid metabolism, phenylalanine metabolism, purine metabolism, arachidonic acid metabolism, and bile acid biosynthesis pathways and may indicate the potential efficacy of the medication in RA. 

Overproduction of oxidants leads to oxidative tissue damage at the molecular level. A growing number of reports have provided evidence that implicates oxidative injury as a major pathogenic mechanism in RA [24–27]. Therefore, protecting joints from oxidative injury may provide a useful therapeutic potential for RA prevention and treatment [28, 29]. Biomarkers related to glycerophospholipid metabolism (e.g., choline and glycerophosphocholine) and fatty acid metabolism (e.g., carnitine, acetylcarnitine, palmitoyl-L-carnitine and palmitic acid methyl ester) were all upregulated in CIA rats (except for carnitine), indicating that RA caused increased lipid catabolism. Uric acid and allantoin also contributed to oxidative injury *in vivo *[30–32]. Uric acid protected the DNA against free-radical damage [33, 34], while allantoin, which was detected and measured in biological fluids and tissues, was produced after uric acid oxidation. Therefore, the downregulation of uric acid and the upregulation of allantoin observed in the model group indicate that oxidative reactions led to serious damage in CIA rats. This observation also demonstrates that urate plays the role of a natural antioxidant related to purine metabolism *in vivo*. Carnitine is required for the transport of long chain fatty acids and acyl coenzyme A derivatives across the inner mitochondrial membrane. Several reports have shown that carnitine has protective effects against oxidative damage [35]. Carnitine not only participates in the metabolism of reactive oxygen species [14] but also plays a role in fatty acid energy metabolism [36, 37]. Other studies have shown that plasma carnitine levels were significantly lower in RA patients than in a control group [38], whereas long chain acylcarnitine levels were higher in RA patients [39]. These results are consistent with the ionic response trends that were observed in the present study. Notably, bile acid promotes the digestion and absorption of fatty acids and has an ameliorating effect on RA [40]. Bile acid biosynthesis (e.g., glycocholic acid and deoxycholic acid) was downregulated in CIA rats, indicating a disruption in fatty acid metabolism. Phenylalanine and tryptophan metabolism was discussed in our previous study of urine metabolomics; the results obtained here were complementary to our earlier study [23].

In summary, 18 potential biomarkers were identified. These markers are mainly associated with glycerophospholipid metabolism, fatty acid metabolism, tryptophan metabolism, linoleic acid metabolism, phenylalanine metabolism, purine metabolism, arachidonic acid metabolism, and bile acid biosynthesis and reveal RA regulating network *in vivo*.

### 3.5. Metabolomic Analysis of HLJDT and Its Treatment Components

**Figure 6:**
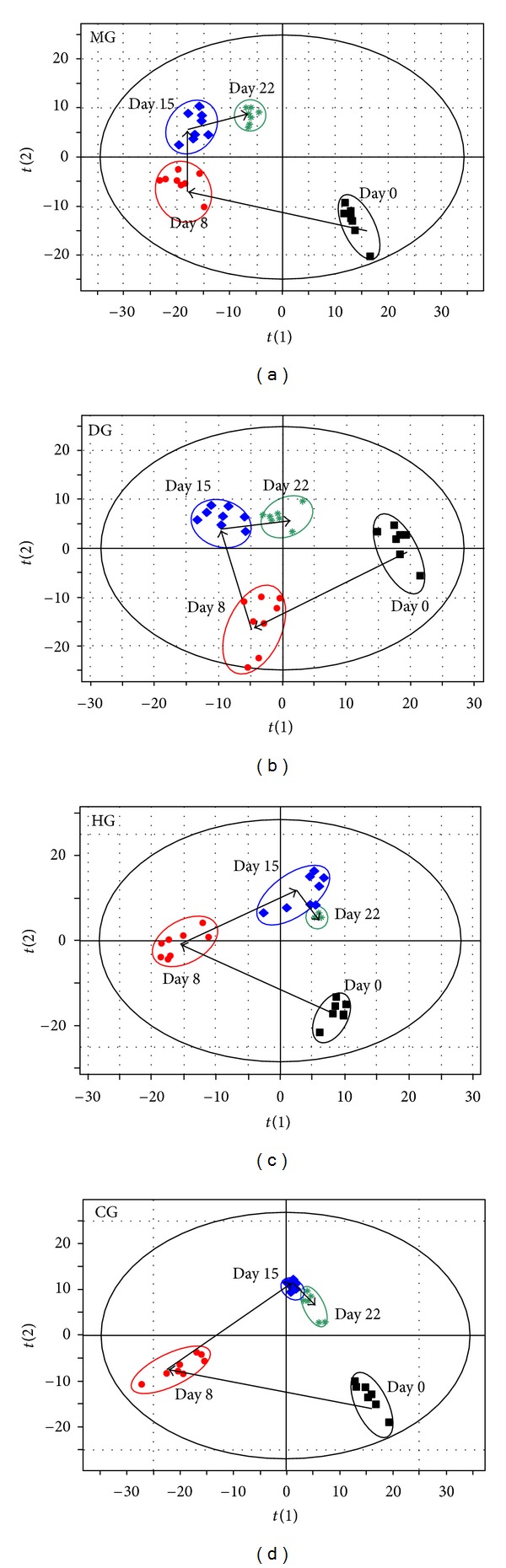
Dynamic PCA scores' plots of plasma metabolites impacted by different groups from day 0 to 22. (a) HLJDT group, (b) dexamethasone group, (c) components group, and (d) model group. HG, HLJDT group; DG, dexamethasone group; CG, components group; MG, model group.

**Figure 7:**
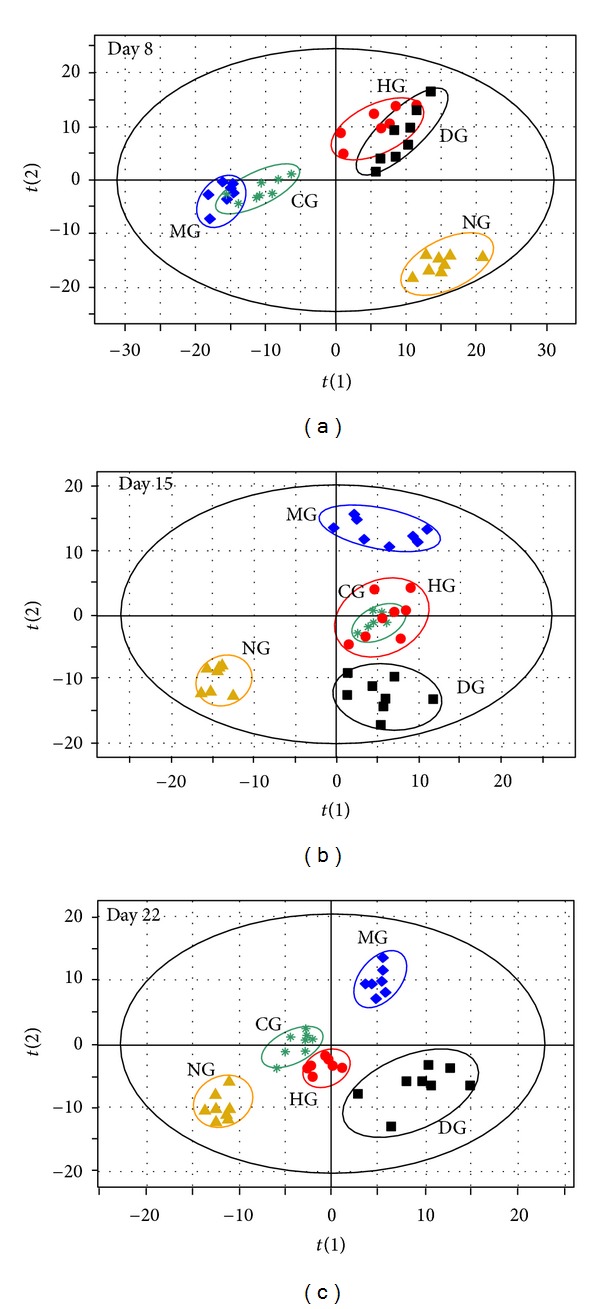
Comparison of PLS-DA scores plots of rat plasma data of different groups on days 8, 15, and 22. (a) Day 8 (*Q* ^2^ *Y*_(cum)_ = 0.777, *R* ^2^ *X*_(cum)_ = 0.428, *R* ^2^ *Y*_(cum)_ = 0.98). (b) Day 15 (*Q* ^2^ *Y*_(cum)_ = 0.796, *R* ^2^ *X*_(cum)_ = 0.41, *R* ^2^ *Y*_(cum)_ = 0.985). (c) Day 22 (*Q* ^2^ *Y*_(cum)_ = 0.804, *R* ^2^ *X*_(cum)_ = 0.439, *R* ^2^ *Y*_(cum)_ = 0.993). NG, normal control group; MG, model group; DG, dexamethasone group; HG, HLJDT group; CG, components group.

PCA, an unsupervised pattern recognition method, was used to observe trends in mean metabolite pattern changes across various time points (Figure 6). PLS-DA, a supervised pattern recognition method, was used to display the metabolic state of CIA rats on days 8, 15, and 22 (Figure 7). The location marked with an arrow in Figure 6 indicates the trend in mean metabolite pattern changes. On day 8, each group's metabolic state had changed from its initial position (day 0). This change indicates that RA had disrupted endogenous substance metabolism and had significantly altered the metabolic fingerprints of the plasma compared to its normal state. From day 8 to day 22, the direction of the trajectory gradually moved towards the initial space. The trajectory then returned to the initial space, indicating a recovery from the disrupted metabolic state. Compared to the model control group, the three drug treatment groups showed better recovery performance from the CIA-induced metabolic state. This result can be observed by comparing the dynamic trajectories in Figures 6 and 7(c). While the dexamethasone group had the advantage of rapid treatment, the toxic side effects of long-term dexamethasone administration led to a metabolic state that deviated from normal rats on day 22. Furthermore, HLJDT and its components resulted in better recovery performance from the CIA-induced metabolic state than dexamethasone on day 22 (Figure 7(c)). 

Nine metabolites were reversed by HLJDT, and 7 were reversed by its components (Table 3). This result indicates that the component group could largely replace the effects of the complete formula. The metabolites that were reversed are primarily involved in phenylalanine metabolism, glycerophospholipid metabolism, fatty acid metabolism, and arachidonic acid metabolism, which indicate that the effectiveness of HLJDT and its components as an RA treatment partially depends on restoring imbalances related to oxidative injury and the arachidonic acid pathway. HLJDT was reported to have protective and therapeutic effects on peripheral inflammation and hepatotoxin-induced liver injuries [41, 42]. *Rhizoma coptidis* and *Radix scutellariae* were responsible for the suppressive effect of HLJDT on eicosanoid generation. Some of their pure components, including baicalein, baicalin, wogonoside, wogonin, coptisine, and magnoflorine, were also shown to inhibit eicosanoid generation in rat macrophages via the arachidonic acid cascade [21]. 

The results from a lipid peroxide assay, antioxidant enzyme activity assays, and metabolomic analysis demonstrate that HLJDT and its components have extensive effects in RA treatment by regulating the pathway disruptions associated with oxidative injury and arachidonic acid metabolism. 

## 4. Conclusions

In this study, metabolomic analysis with LC-Q-TOF-MS was used to profile RA-related metabolic changes in the plasma and to investigate the interventional mechanisms of HLJDT and its components. After multiple levels of statistical analysis, 18 significant biomarkers (11 metabolites detected in the positive mode and 7 metabolites detected in the negative mode) were identified. These biomarkers are primarily involved in glycerophospholipid metabolism, fatty acid metabolism, tryptophan metabolism, linoleic acid metabolism, phenylalanine metabolism, purine metabolism, arachidonic acid metabolism, and bile acid biosynthesis. Potential biomarkers-related glycerophospholipid metabolism and fatty acid metabolism, namely, carnitine, acetylcarnitine, allantoin, uric acid, choline, and glycerophosphocholine, appear to have diagnostic and/or prognostic values for RA and require further investigation in clinical studies. Using the potential biomarkers identified in this study as a screening index, we hypothesize that HLJDT and its components can limit the pathological process of RA by partially reversing metabolite levels and regulating pathway disruptions. The metabolomic results presented here provide a systemic view of the development and progression of RA as well as a theoretical basis for the prevention or treatment of RA. 

## Figures and Tables

**Table 1:** Effects of HLJDT and its components on MDA levels and antioxidant enzymes' activities on day 22.

Groups	MDA (nmol/mL)	SOD (U/mL)^a^	GSH-Px (U/mL)^b^
Normal control	2.71 ± 0.34	4.56 ± 0.24	7.72 ± 0.71
Model control	4.98 ± 0.53^##^	2.95 ± 0.22^##^	4.97 ± 1.56^#^
Dexamethasone	2.62 ± 0.28**	4.23 ± 0.33**	7.69 ± 1.38*
HLJDT	2.99 ± 0.38**	4.74 ± 0.29**	7.30 ± 1.13*
Components of HLJDT	2.91 ± 0.42**	5.27 ± 0.22**	6.82 ± 0.91*

SOD: superoxide dismutase; GSH-Px: glutathione peroxidase. *P < 0.05, **P < 0.01, ^#^ *P* < 0.05, and ^##^ *P* < 0.01 (^#^: compared with normal control group; *: compared with model control group).

^a^One unit of SOD activity is defined as amount of SOD when SOD inhibition ratio reaches 50% in 1 mL reaction solution.

^b^One unit of GSH-Px activity is defined as amount of enzyme required to degrade 1 *μ*mol/L of GSH per min subtracting nonenzymatic reaction at 37°C.

**Table 2:** Potential biomarkers in response to RA and their metabolic pathways.

Mode	Number	*t*_*R*_/min	*m*/*z*	Formula	Identification	Fold^a^	*P* value^a^	Related pathway
	1	1.16	104.1076	C_5_H_14_NO	Choline^b^	26.11	0.000	Glycerophospholipid metabolism
	2	1.19	162.1129	C_7_H_15_NO_3_	Carnitine^b^	−0.84	0.000	Fatty acid metabolism, oxidative injury
	3	1.20	258.1108	C_8_H_21_NO_6_P	Glycerophosphocholine^c^	25.39	0.000	Glycerophospholipid metabolism
	4	1.73	204.1235	C_9_H_17_NO_4_	Acetylcarnitine^c^	0.819	0.006	Fatty acid metabolism, oxidative injury
	5	3.06	166.0869	C_9_H_11_NO_2_	L-Phenylalanine^b^	0.81	0.005	Phenylalanine metabolism
ESI(+)	6	5.07	180.0661	C_9_H_9_NO_3_	Hippuric acid^b^	25.27	0.007	Phenylalanine metabolism
	7	5.30	194.0820	C_10_H_11_NO_3_	Phenylacetylglycine^c^	−0.93	0.004	Phenylalanine metabolism
	8	10.13	357.2796	C_24_H_36_O_2_	DHA ethyl ester^c^	3.19	0.002	Alpha linolenic acid and linoleic acid metabolism
	9	14.89	400.3427	C_23_H_45_NO_4_	Palmitoyl-L-carnitine^c^	0.57	0.003	Fatty acid metabolism
	10	16.58	305.2481	C_20_H_32_O_2_	Arachidonic acid^b^	0.94	0.002	Arachidonic acid metabolism
	11	16.79	271.2637	C_17_H_34_O_2_	Palmitic acid methyl ester^c^	4.09	0.000	Fatty acid metabolism

	12	1.24	157.0361	C_4_H_6_N_4_O_3_	Allantoin^b^	2.13	0.000	Purine metabolism, oxidative injury
	13	1.74	167.0207	C_5_H_4_N_4_O_3_	Uric acid^b^	−1.16	0.004	Purine metabolism, oxidative injury
	14	2.19	219.0775	C_11_H_12_N_2_O_3_	5-Hydroxy tryptophan^b^	0.99	0.008	Tryptophan metabolism
ESI(−)	15	4.51	203.0831	C_11_H_12_N_2_O_2_	L-Tryptophan^b^	1.61	0.003	Tryptophan metabolism
	16	8.34	212.0025	C_8_H_7_NO_4_S	Indoxyl sulfate^c^	1.27	0.003	Tryptophan metabolism
	17	6.21	464.3024	C_26_H_43_NO_6_	Glycocholic acid^c^	−0.38	0.022	Bile acid biosynthesis
	18	10.14	391.2855	C_24_H_40_O_4_	Deoxycholic acid^c^	−0.44	0.046	Bile acid biosynthesis

^a^Fold changes (calculated as log_2_ (average peak intensity of model group/average peak intensity of normal control group) and *P* value compared with normal control group on day 22.

^b^Metabolites validated with standards.

^c^Metabolites putatively annotated.

**Table 3:** Summary of potential biomarkers in HLJDT and its components' groups on day 22.

Biomarkers	HLJDT		Components of HLJDT	
	Fold^a^	*P* value^a^	Fold^b^	*P* value^b^
Choline	−20.24	0.041	—	—
Carnitine	0.40	0.000	0.18	0.003
Glycerophosphocholine	−19.87	0.001	−16.79	0.009
Acetylcarnitine	−0.76	0.003	−0.15	0.002
L-Phenylalanine	−0.28	0.012	−0.19	0.043
Hippuric acid	−20.17	0.004	−18.87	0.006
Palmitoyl-L-carnitine	−0.56	0.002	−0.01	0.022
Allantoin	−0.12	0.018	—	—
Arachidonic acid	−0.24	0.002	−0.14	0.003

^a^Fold changes calculated as log_2_ (average peak intensity of HLJDT group/average peak intensity of model group) and *P* value compared with model group on day 22.

^b^Fold changes calculated as log_2_ (average peak intensity of components of HLJDT group/average peak intensity of model group) and *P* value compared with model group on day 22.

## Abbreviations

RA: Rheumatoid arthritis
CIA: Collagen-induced arthritis
LC-Q-TOF-MS: Liquid chromatography quadrupole time-of-flight mass spectrometry
HLJDT: Huang-Lian-Jie-Du-Tang formula
NG: Normal group
MG: Model group
DG: Dexamethasone group
HG: HLJDT group
CG: Components group
TCM: Traditional Chinese medicine
PCA: Principal component analysis
PLS-DA: Partial least squares discriminant analysis
GSH-Px: Glutathione peroxidase
SOD: Superoxide dismutase
MDA: Malondialdehyde
TBA: Thiobarbituric acid.
